# Photoperiodic Effects on Seasonal Physiology, Reproductive Status and Hypothalamic Gene Expression in Young Male F344 Rats

**DOI:** 10.1111/jne.12241

**Published:** 2015-01-26

**Authors:** F M Tavolaro, L M Thomson, A W Ross, P J Morgan, G Helfer

**Affiliations:** Rowett Institute of Nutrition and Health, University of AberdeenAberdeen, UK

**Keywords:** photoperiod, F344 rat, body weight, reproduction, hypothalamic gene expression

## Abstract

Seasonal or photoperiodically sensitive animals respond to altered day length with changes in physiology (growth, food intake and reproductive status) and behaviour to adapt to predictable yearly changes in the climate. Typically, different species of hamsters, voles and sheep are the most studied animal models of photoperiodism. Although laboratory rats are generally considered nonphotoperiodic, one rat strain, the inbred Fischer 344 (F344) rat, has been shown to be sensitive to the length of daylight exposure by changing its physiological phenotype and reproductive status according to the season. The present study aimed to better understand the nature of the photoperiodic response in the F344 rat. We examined the effects of five different photoperiods on the physiological and neuroendocrine responses. Young male F344 rats were held under light schedules ranging from 8 h of light/day to 16 h of light/day, and then body weight, including fat and lean mass, food intake, testes weights and hypothalamic gene expression were compared. We found that rats held under photoperiods of ≥ 12 h of light/day showed increased growth and food intake relative to rats held under photoperiods of ≤ 10 h of light/day. Magnetic resonance imaging analysis confirmed that these changes were mainly the result of a change in lean body mass. The same pattern was evident for reproductive status, with higher paired testes weight in photoperiods of ≥ 12 h of light/day. Accompanying the changes in physiological status were major changes in hypothalamic thyroid hormone (*Dio2* and *Dio3*), retinoic acid (*Crabp1* and *Stra6*) and Wnt/β-Catenin signalling genes (*sFrp2* and *Mfrp*). Our data demonstrate that a photoperiod schedule of 12 h of light/day is interpreted as a stimulatory photoperiod by the neuroendocrine system of young male F344 rats.

Seasonal or photoperiodically sensitive animals make profound anticipatory changes in physiology (growth, food intake and reproductive status) and behaviour to adapt to predictable yearly changes in the climate. As such, they provide excellent model systems for studying neuroendocrinology, as well as long-term adaptive changes in the hypothalamus.

Over the past decade, studies of a variety of seasonal species, including mammals and birds, have helped us to understand the primary effects of photoperiod on the neuroendocrine hypothalamus. Initiated by work conducted in the Japanese quail, it has been shown that the seasonal cycles in reproduction are critically dependent upon the regulation of thyroid hormone metabolism in the hypothalamus (1,2). These observations have been extended to mammals, including hamsters, sheep and rats, and the results suggest that, in addition to the traditional hypothalamo-pituitary axes, an ‘inverse neuroendocrine axis’ exists, where the pituitary controls the hypothalamus (3–5). It is now generally recognised that, in mammals, photoperiod regulates thyroid-stimulating hormone production in the pars tuberalis of the pituitary via nocturnal melatonin secretion from the pineal gland. Thyroid-stimulating hormone, in turn, regulates hypothalamic thyroid hormone activation by controlling deiodinase enzymes (Dio2 and Dio3) in the ependymal cells lining the third ventricle of the hypothalamus. The resulting switch in local thyroid hormone availability is assumed to comprise the basis of long-term seasonal changes in growth, food intake and reproductive status. Although this pathway is highly conserved between vertebrates, unlike in mammals, these mechanisms are independent of melatonin in birds (6,7). The photoperiodic variations in thyroid hormone metabolism are not exclusive to seasonal animals because Ono *et al*. (8) reported photoperiod and melatonin-driven changes in Dio2 and Dio3 expression in nonphotoperiodic strains of laboratory mice. In addition to thyroid hormone signalling, another important component of the photoperiodic response is hypothalamic retinoic acid signalling, which has also been associated with profound changes in growth and energy balance (9).

Seasonal changes in physiology and reproduction are robust innate processes that require long-term changes so that the animals can anticipate and adapt accordingly in advance of predictable yearly changes in the environment. Yet even small mammals that do not display circannual rhythms, as a result of their short life-span, show pronounced seasonal cycles in body mass, food intake and reproduction (6). As such, typical animal models for the study of photoperiodism often comprise long-day breeding seasonal rodents because they show rapid and dramatic adaptations in their morphology, physiology and behaviour to changes in photoperiod. For example, different species of hamster and vole suppress reproduction, food intake and somatic growth in inhibitory short photoperiods and increase these parameters in stimulatory long photoperiods. The critical photoperiod for the switch between inhibitory and stimulatory photoperiods is species-dependent (10).

It is only comparatively recently that research has been directed towards using laboratory rats for studying seasonal physiology and reproduction. This is because laboratory rats are generally characterised as nonseasonal animals in that they reproduce year-round and have only little or no reproductive response to a short photoperiod. However, some strains of rats are photoresponsive but the sensitivity and magnitude of the photoperiodic response varies (11,12), although, after specific manipulations, such as olfactory bulbectomy (13) or treatment with testosterone implants to increase steroid negative-feedback (14,15), most laboratory strains of rats have the potential to respond to photoperiod. Nevertheless, the strong advantage of their use is the availability of a wealth of genetic information in publicly available databases and in the literature, as well as the molecular probes and tools that are lacking for the study of other seasonal species.

One rat strain, the inbred Fisher (F) 344 rat, displays a strong physiological and reproductive response to different photoperiods (12). In young F344 rats, a long-day exposure (more than 14 h light/day) induces a long-day phenotype, which is characterised by body weight gain (in both lean and fat mass) and increased food intake, as well as testes recrudescence (i.e. an increase in testis weight and size indicating an increase in fertility). Under short-day exposure, F344 rats show a reduction in body weight (both lean and fat mass), a reduced voluntary food intake, and a reduction in testis weight and size, indicating reproductive quiescence (12,16,17). This strong innate response to changes in the light/dark cycle is dependent on melatonin (17,18) and is also reflected by changes in key hypothalamic genes for photoperiodic regulation (17,19). However, the magnitude of this response varies between the substrains of F344 rats. Although F344/NHsd show a robust reduction in growth and food intake, F344/NCr show only a comparatively small (but still significant) reduction in growth and food intake when exposed to short days (20).

Although a wealth of research data from the last 40 years exists describing the responses of hamsters and voles to photoperiod, there is only limited information available on the seasonal responses of the F344 rat. To better understand the results obtained using the F344 rat and to be able to compare these with other photoperiodic model systems and nonseasonal animals, more information is required on the consequences of photoperiodic history and the response of this strain to different artificial photoperiods. The present study therefore aimed to test whether there are critical photoperiod conditions of light duration required for the full photoperiodic response in F344 rats and also whether the magnitude of the response is related to these conditions. Comparing body mass (including lean and fat mass), food intake, testes weights and hypothalamic gene expression under five different photoperiods, we found a general pattern emerged: photoperiods of ≤ 10 h of light/day resulted in an inhibition of growth, food intake and reproduction, as well as hypothalamic gene expression, whereas all other photoperiods resulted in a stimulatory response in these parameters.

## Materials and methods

### Animals and tissue collection

All animal experiments were approved by the local ethics committee at the Rowett Institute of Nutrition and Health, University of Aberdeen (approval number SA11/02E) and licensed under the Animals (Scientific Procedures) Act, 1986 (project licence number PPL60/3615).

Male Fischer 344/NHsd (from hereon referred to as F344) rats were obtained from Harlan Sprague Dawley Inc. (Indianaplis, IN, USA) at 3–4 weeks old. Rats were acclimatised for 10 days under an L12 (12 h light/day) photocycle and then randomly divided into weight-matched groups of eight rats each and transferred to different photoschedule rooms: L8 (8 h light/day), L10 (10 h light/day), L12 (12 h light/day), L14 (14 h light/day) and L16 (16 h light/day). In all rooms, the original lights-on time stayed the same; therefore, photoperiods were changed by shortening or lengthening the lights-off time. In their respective photoperiod room, rats were single housed in standard rat cages (type RC2/f; NKP Cages, Coalville, UK) with a plastic tunnel and shredded paper as enrichment and G6 woodchip bedding (DBM, Scotland Ltd, Broxburn, UK). Food [CRM (P) Rat and Mouse Breeder and Grower, standard pelleted diet; Special Diet Services, Witham, Essex, UK] and water was provided *ad lib*. and, besides the light/dark schedule, all other conditions were kept constant (temperature: 21 ± 2 °C; humidity: 55 ± 10%). As a result of room availability, the study was performed in two parts. Animals were weight- and age-matched for both parts and each part included a L12 group to ensure comparability (the first part included L8, L12 and L16; the second part included L10, L12 and L14). For all rats, body weights were measured three times weekly and food intakes were recorded daily at the beginning of the light phase. Whole body composition (total fat and lean) of conscious rats was quantified by magnetic resonance imaging (MRI) (EchoMRI, Houston, TX, USA) once a week, as described previously (21). After 4 weeks in their respective photoperiods, rats were anaesthetised using isoflurane inhalation and killed by decapitation at Zeitgeber time 3 (i.e. 3 h after lights on). Before killing, animals were given random numbers to ensure blind analyses for the photoperiodic conditions. Hypothalamic tissue was immediately removed on wet ice, frozen on dry ice and stored at −80 °C. Testes were dissected and paired testes weight was recorded.

### Isolation of RNA and quantitative real-time polymerase chain reaction (PCR) analysis

Total RNA was isolated from 60 to 80 mg of hypothalamic tissue using an RNeasy Mini Kit with on-column DNase treatment in accordance with the manufacturer's instructions (Qiagen, Valencia, CA, USA). The quantity and quality of the resulting RNA was measured using a NanoDrop ND-1000 spectrophotometer (Thermo Scientific, Wilmington, DE, USA) and a Bioanalyzer 2100 (Agilent Technologies, Santa Clara, CA, USA). One microgram of total RNA was copied into cDNA using a High-Capacity cDNA reverse transcription kit (Applied Biosystems, Foster City, CA, USA). Five nanograms of the resulting cDNA was then analysed by quantitative real-time PCR (qPCR) using a QuantiFastTM SYBR Green PCR kit (Qiagen) with the Thermal Cycler 7500 Fast Real Time PCR System (Applied Biosystems). The primers used were validated QuantiTect Primer Assay obtained from Qiagen: *β-actin* (Rn_Actb_1_SG), *Crabp1* (Rn_Crabp1_1_SG), *Dio2* (Rn_Dio2_2_SG), *Dio3* (Rn_Dio3_1_SG), *Mfrp* (Rn_Mfrp_3_SG), *sFrp2* (Rn_Sfrp2_1_SG) and *Stra6* (Rn_Stra6_2_SG). The reaction conditions were: amplification 5 min at 95 °C, 40 cycles of 10 s at 95 °C, 30 s at 60 °C and melting curve analysis 15 s at 95 °C, 1 min at 65 °C, 15 s at 95 °C and 15 s at 60 °C. Each sample was run three times and each PCR plate included a negative control reaction without template. The *β-actin* housekeeping gene was used as reference for the relative quantification of *Crabp1*, *Dio2*, *Dio3*, *Mfrp2*, *sF*rp2 and *Stra6* calculated based on the 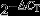 method.

### Statistical analysis

Data were analysed by one-way anova or two-way repeated measures (RM) anova (photoperiod × time interaction) followed by Tukey's honestly significant difference post-hoc test for pairwise comparison as appropriate, using sigmaplot, version 12.0 (Systat Software Inc., Chicago, IL, USA). No significant difference was found between L12 from the first and the second part of the study (P* *=* *0.884); therefore, L12 from the first part of the study was used for the final data analysis. The results are presented as the mean ± SEM or fold change (± SEM) relative to L12 for physiological data and qPCR analysis, respectively. P* *<* *0.05 was considered statistically significant.

## Results

### Effect of photoperiod on physiology

#### Body weight, food intake and testes weights

**Figure 1:**
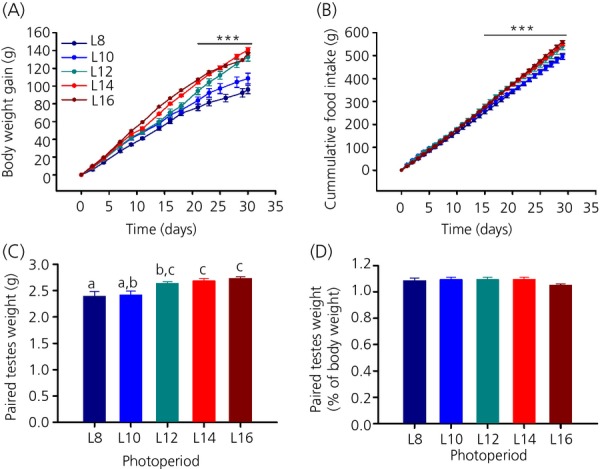
Effect of photoperiod on body weight, food intake and testes weight. (a) Body weight gain and (b) cumulative food intake was significantly lower in L8 and L10 compared to L12, L14 and L16. (c) Paired testes weights of male F344 rats under different photoperiods. For each group, different lowercase letters above bars indicate significant differences (P < 0.05) between photoperiod groups. (d) Paired testes weights as a percentage of body weight. Data are the mean ± SEM; *** P* *<* *0.001.

Significant differences were found among photoperiod groups with respect to body weight gain (two-way RM anova, P* *<* *0.001; Fig.1a), cumulative food intake (two-way RM anova, P* *<* *0.001; Fig.1b) and paired testes weight (one-way anova, P* *<* *0.001; Fig.1c). In all three measures, L8 and L10 were smaller than those of L12, L14 and L16 after 4 weeks in a photoperiod. A pairwise comparison of body weight gain showed that L8 and L10 were significantly different from L12, L14 and L16, although no significant difference could be found between L8 and L10 or L12, L14 and L16 after 4 weeks in a photoperiod. Initially, for the first 18 days, L12 followed the lower incline of L8 and L10 but, after 21 days in a photoperiod, L12 rats increased their body weight to match**.** L14 and L16 rats (Tukey's test, P* *<* *0.05; Fig.1a). No difference was detectable in cumulative food intake between L8 and L10 or between L12, L14 and L16. L8 and L10 rats ate significantly less than L12, L14 and L16 rats after 15 days in a photoperiod and this trend continued until 30 days (Tukey's test, P* *<* *0.05; Fig.1b). The same pattern was evident with regard to testes weight (Fig.1c). A pairwise comparison showed that there was no significant difference between L8 and L10, nor between L12, L14 and L16 in paired testes weight. In this case, no significant difference was found between L10 and L12 (Tukey's test, P* *=* *0.087), although L10 was significantly lower compared to L14 and L16 (Tukey's test, P* *=* *0.022 and 0.006, respectively). Paired testes weight of L8 was also significantly lower compared to L12, L14 and L16 (Tukey's test, P* *<* *0.05; Fig.1c). The effects on testes size were in proportion to body weight because no difference was detectable between the means of the photoperiod groups when assessing paired testes weights as a percentage of body weight (one-way anova, P* *=* *0.306; Fig.1d).

#### Body composition

**Figure 2:**
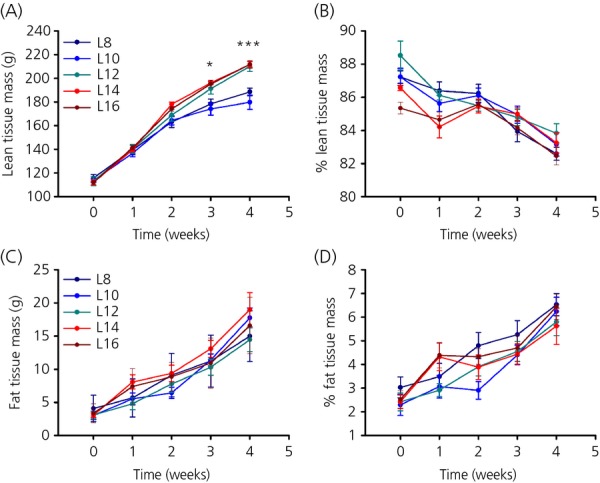
Effect of photoperiod on body composition. (a) Total lean tissue mass in g and (b) lean tissue mass as a percentage of body weight of F344 rats held under different photoperiods (c) Total fat tissue mass (g) and (d) fat tissue mass as a percentage of body weight in F344 rats held under different photoperiods. Data are the mean ± SEM; P < 0.05, *** P < 0.001.

To investigate the body composition of F344 rats held under different photoperiods, rats were subjected to weekly MRI scans (week 0 refers to the day before the rats were put into photoperiods; week 4 refers to the day before killing). As with body weight, rats on L8 and L10 gained less lean mass than rats on L12, L14 and L16 (two-way RM anova, P* *<* *0.001; Fig.2a). The effect in lean mass was in proportion to body weight. With time, the percentage of lean mass decreased from approximately 87% to 83% independent of photoperiod (Fig.2b), whereas the percentage of fat mass increased from approximately 2.5% to 6.1%, again independent of photoperiod (Fig.2d). Total fat mass increased with time (two-way RM anova, P* *<* *0.001), although there was no detectable effect of photoperiod (P* *=* *0.512; Fig.2c).

### Effect of photoperiod on hypothalamic gene expression

A wide range of genes that are photoperiodically regulated in the hypothalamus have been identified in F344 rats, including genes involved in thyroid hormone signalling, retinoic acid signalling and Wnt/β-Catenin signalling (17). In the present study, we selected two representative examples of each of these pathways and investigated their response to the five different photoperiods by qPCR. Although there were some gene specific differences (described in detail below), the general pattern for all genes investigated was similar and comparable to the results of the physiological response. L8 and L10 always appeared to form one group and, at the same time, L12, L14 and L16 appeared to form one group.

#### Thyroid hormone signalling

**Figure 3:**
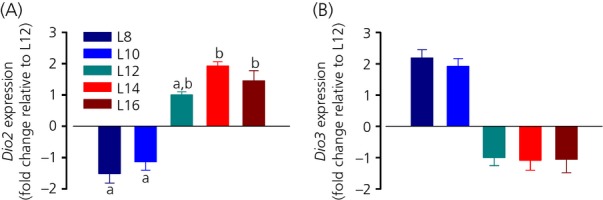
Effect of photoperiod on hypothalamic thyroid hormone signalling. (a) *Dio2* and (b) *Dio3* mRNA expression under five different photoperiods. For each group, different lowercase letters above bars indicate significant differences (P < 0.05) between photoperiod groups. Data are shown as fold changes relative to L12 (± SEM). Note that, for clarity, L12 has been set to −1 in (b).

The expression level of *Dio2* mRNA was lower in F344 rats exposed to L8 and L10 than in rats exposed to L12, L14 and L16 (one-way anova, P* *<* *0.001; Fig.3a). A pairwise comparison also did not find a significant difference between L8 and L12 or L10 and L12 in this case (Tukey's test, P* *=* *0.460 and 0.983, respectively). By contrast to *Dio2*, the expression of *Dio3* was highest under L8 and L10 and lowest under L12, L14 and L16 (one-way anova, P* *=* *0.013; Fig.3b). Here, a pairwise multiple comparison procedure (Tukey's test) did not identify any significant difference between the individual photoperiod groups.

#### Retinoic acid signalling

**Figure 4:**
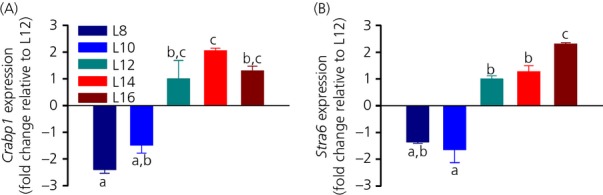
Effect of photoperiod on hypothalamic retinoic acid signalling genes. (a) *Crabp1* and (b) *Stra6* mRNA expression under five different photoperiods. For each group, different lowercase letters above bars indicate significant differences (P* *<* *0.05) between photoperiod groups. Data are shown as fold changes relative to L12 (± SEM).

The levels of *Crabp1* and *Stra6* mRNA were similarly regulated by photoperiod, with low levels expressed under L8 and L10 and high levels under L12, L14 and L16 (one-way anova, P* *<* *0.001 for both; Fig.4). A pairwise comparison did not detect a significant difference between L8 and L10 (Tukey's test, P* *=* *0.472) or L12, 14 and L16 (Tukey's test, P* *=* *0.134 and 0.909, respectively) in *Crabp1* mRNA expression. No significant difference was found between L10, L12 and L16, although L10 was significantly lower compared to L14 (Tukey's test, P* *=* *0.009; Fig.4a). Figure4(b) shows *Stra6* mRNA expression. Again, L8 and L10 were not significantly different from each other (Tukey's test, P* *=* *0.472) and L12 and L14 were not significantly different from each other (Tukey's test, P* *=* *0.134), although L16 was higher than any other group, with a 2.3-fold increase compared to L12 (Tukey's test, P* *=* *0.016).

#### Wnt/β-Catenin signalling

**Figure 5:**
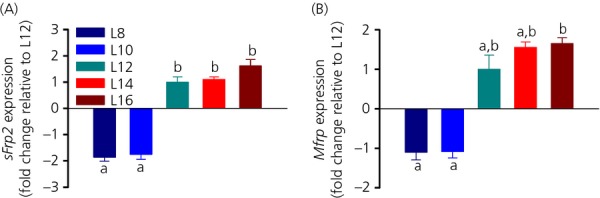
Effect of photoperiod on hypothalamic Wnt/β-Catenin signalling. (a) *sFrp2* and (b) *Mfrp* mRNA expression under five different photoperiods. For each group, different lowercase letters above bars indicate significant differences (P < 0.05) between photoperiod groups. Data are shown as fold changes relative to L12 (± SEM).

The Wnt/β-Catenin signalling genes *sFrp2* and *Mfrp* were also strongly influenced by photoperiod with similar expression patterns. *sFrp2* mRNA levels were approximately 1.8-fold lower under L8 and L10 compared to L12, 14 and L16 (one-way anova, P* *<* *0.001; Fig.5a). The expression level of *Mfrp* was similarly affected by photoperiod, although the effect was less dramatic (one-way anova, P* *=* *0.006; Fig.5b). A pairwise comparison only found a significant difference between L8 and L16, as well as L10 and L16 (Tukey's t6est, P* *=* *0.043 and 0.039, respectively). No significant difference was found between the means of the other photoperiod groups.

## Discussion

In the present study, we show that juvenile males of the F344 strain of laboratory rats display a robust but varied response to a range of photoperiods with respect to their body weight, food intake, gonadal growth and key genes for the photoperiodic regulation. These results are consistent with previous reports on the photoperiod sensitivity of young F344 rats (12,16,17,22) and extend them to show that photoperiods of ≤ 10 h of light/day (L8 and L10) result in a reduction of growth, food intake and an inhibitory response in hypothalamic gene expression, whereas photoperiods of ≥ 12 h of light/day (L12, L14, L16) result in a stimulatory physiological and gene expression response. Also, there was no increase in response as the length of the light period was increased beyond the threshold of 12 h of light.

F344 rats are increasingly used as a model for studying photoresponsiveness (17,23–25), although more information is required on the consequences of photoperiodic history and the responses of this strain to changing photoperiod to be able to compare the results with those obtained from other well-studied photoperiodic species. We aimed to design the present study to reflect the experimental design of a typical photoperiodic study (i.e. young F344 rats were abruptly transferred to photoperiod rooms, a practise commonly used in laboratory settings). Moreover, in most photoperiod studies, animals are tested either in short (≤ 10 h of light/day) and/or long (≥ 14 h of light/day) photoperiods and thus no inferences can be made on intermediate photoperiods (12 h light/day; L12). Therefore, by designing the study to acclimatise rats under L12 and transferring them from L12 to other photoperiods, we were able to investigate the question whether L12 generates inhibitory (short day) or stimulatory (long day) responses in terms of seasonal physiology and hypothalamic gene expression. F344 rats are the most widely used inbred strain of rats in research (26). In general, laboratory rats are held and commonly tested under a L12 photoperiod; therefore, it is important to understand whether L12 is construed as an inhibitory or stimulatory photoperiod by the neuroendocrine system because this will impact on the reproductive axis, endocrine and metabolic state, which can affect most organ systems.

Analysis of body weight gain in the five photoperiod groups in young F344 rats showed that abrupt changes in photoperiod resulted in the expected reduction of body mass for day lengths < 12 h and also the expected increase of body mass for day lengths > 12 h (16,22). After 4 weeks in a photoperiod, no difference was observed in body weight gain in L8 and L10 rats that followed the slower rate in growth of short-day animals. On the other hand, L12, L14 and L16 rats showed a faster rate of growth typically seen in long-day animals. This pattern was also evident with respect to cumulative food intake, indicating that L12 rats followed a long-day response with respect to these physiological parameters. Notably, for the first third of the study, L12 rats had the lower growth rate of L8 and L10 rats, but, after approximately 3 weeks in a photoperiod, the body weight gain of L12 rats resembled that of L14 and L16 rats.

Previously, Shoemaker *et al*. (22) reported an inhibitory effect of body weight on F344 rats held under L12 similar to that of rats held under L8 until approximately 8–10 weeks after weaning. However, when comparing body weight as a percentage of L16 rats, 8-week-old rats (comparable to the age of the rats in the present study) held under L12 have a higher body weight than rats held under L8. Unfortunately, no statistical differences between L8 and L12 were reported and therefore no direct comparison can be made with the present study. Importantly, the present study also differed in respect of F344 rats being born and raised under an L12 photoperiod, whereas, in the study by Shoemaker *et al*. (22), rats were kept under L16 from gestation (22). Somatic growth in Siberian hamsters is strongly influenced by the photoperiodic history experienced during gestation and after birth (27,28), although it remains to be determined whether this is also the case in F344 rats.

A closer analysis of body composition revealed that the differential gain in body weight between F344 rats on L8 and L10 compared to those on L12, 14 and L16 was mainly the result of changes in lean body mass. Absolute lean tissue mass resembled that of body weight gain in such a way that there were two distinct responses: a short-day response of L8 and L10 rats and a long-day response of L12, L14 and L16 rats. However, photoperiodic exposure showed no significant difference in total fat tissue mass. Thus, the voluntary increases in food consumption by the F344 rats under longer photoperiod conditions lead to an increase in somatic growth rather than a gain in adiposity (20). In other seasonal rodents, the change in body weight is predominantly the result of a change in fat mass (10), although more recent studies indicate that Siberian hamsters kept under short-day photoperiods may not only reduce fat mass, but also reduce the amount of lean mass (29–31). This includes a reduction in liver, kidney (30), spleen and muscle mass (31), although no photoperiodic change in bone morphology has been observed (32).

When analysing the body composition of the F344 rats relative to body weight, no difference in the photoperiod groups was evident with respect to lean and fat mass, whereas, over time, lean mass decreased at the same time as fat mass increased indicating that, in the growing rat, a greater portion of body mass was being deposited as fat mass.

To assess photoperiodic changes in reproduction, we recorded paired testes weight at the time of killing and found that, concordant with previous studies, young male F344 rats are reproductively sensitive to photoperiod (12,16). After 4 weeks in a photoperiod, rats held under L8 and L10 showed a lower testicular growth than rats held under L12, L14 and L16 photoperiods. The photoperiodic effects on testicular growth were in proportion to body weight because no difference was detectable between the photoperiod groups when assessing paired testes weights relative to body weight.

Next, we investigated the relative expression levels of key genes involved in thyroid hormone signalling, retinoic acid signalling and Wnt/β-Catenin signalling in the five different photoperiods in the hypothalamus of young male F344 rats. Genes from these pathways are known to exhibit robust responses to photoperiod (5,17,33) and, because photoperiodic differences in the expression of these genes change in direct proportion to the time spent in photoperiod (17), we hypothesised that the photoperiodic response in body composition and reproduction would also be reflected in hypothalamic gene expression.

From the local thyroid hormone pathway, by far the best characterised in the hypothalamus of seasonal species (6), we investigated *Dio2* and *Dio3*, genes encoding key enzymes in photoperiodic regulation. *Dio2*, which catalyses the conversion of the prohormone thyroxine into the bioactive thyroid hormone triiodothyronine, increased with increasing light duration in photoperiods, with the highest levels of *Dio2* under L14 and L16. These findings are in accordance with previous findings in F344 rats (17,19), as well as with those from other photoperiodic rodents (34–38). From these, only one study looked at photoperiods other than short day (L8 in the present study) and long day (L16 in the present study). In the common vole, an intermediate photoperiod (L12 in the present study) resulted in a reduced expression of *Dio2* similar to short-day levels (38), whereas, in F344 rats, *Dio2* expression in L12 was more similar to long day. However, no significant difference was observed between L8 or L10 and L12, and no significant difference was found between L14 or L16 and L12 indicating that *Dio2* in the hypothalamus of F344 rats held under L12 exhibits an intermediate state rather than a clear short-day or long-day profile. The other thyroid hormone metabolising enzyme shown to be under photoperiodic control in seasonal rodents is *Dio3*, a gene encoding an enzyme that regulates triiodothyronine inactivation (3,34,39,40). As reported previously in F344 rats, the expression of *Dio3* was higher with shorter day-length (17), although the fold change differences in general were less pronounced than was the case for *Dio2* expression. In the common vole, the *Dio3* expression level in L12 was intermediate compared to short and long days (38). The results reported for the regulation of hypothalamic thyroid hormone metabolism in seasonal animals indicate how important these are in seasonal physiology, although much of the data presented indentify species-specific variability (6). Although there is a reciprocal regulation of *Dio2* and *Dio3* in the F344 rats triggered by pars tuberalis-derived thyroid-stimulating hormone (5), in the common vole, the deiodinase enzymes are not expressed at the same time (38), which could account for the differing expression profiles reported in the present study. Additionally, the voles were bred under a light L14 schedule and, as noted previously, the photoperiodic history of the animals will likely influence the photoperiodic response (27,28).

In accordance with our previous findings, both retinoic acid signalling related genes showed strong photoperiodic control (33). *Stra6*, a gene encoding the retinol receptor, and *Crabp1*, which is involved in RA catabolism (9), increased with an increasing day light duration. Similar to *Dio2*, for both genes, L8 and L10 formed a mutual group. L12 appears to have escaped again, revealing a clearly defined short-day or long-day response and was neither significantly different to L8 and L10 or L14 and L16. Comparable photoperiodic differences in retinoic acid-related genes have been shown in Siberian hamster (41–43), although none of those studies investigated photoperiods other than short or long days.

So far, photoperiodic regulation of the Wnt/β-Catenin pathway has only been studied in F344 rats (5,33). Of the Wnt-related signalling genes, we investigated two regulators of the canonical Wnt pathway (44). *sFrp2* expression was low in L8 and L10 and high in L12, L14 and L16. The differences in fold change of *Mfrp* mRNA levels were less distinct than for the other genes studied; however, the general pattern was maintained. L8 and L10 formed a mutual group with low expression and L12, L14 and L16 formed a mutual group exhibiting a higher expression, although no significant difference could be found in L8, L10, L12 and L14.

Although the data presented for hypothalamic gene expression in F344 rats do not show a clearly defined intermediate or long-day response in L12 in all the genes investigated, we only found slight differences and a trend of L12 gene expression towards a long-day profile was evident. Hence, in combination with the observed physiological changes, the gene expression findings infer that young male F344 rats born and raised under L12 will interpret L12 as a long day, and thus a stimulatory day length.

F344 rats provide great potential to complement the information gained from more typical common model species, such as the hamster and vole, used in the study of photoperiodism. Given that growth, reproductive physiology and neuroendocrinology have been well studied in laboratory rats, and given also that rats are more accessible via a range of molecular techniques (e.g. validated qPCR primers as used in the present study) than other photoperiodic species, the use of rats simplifies both the experimental design and the laboratory work. In addition, one strong advantage to using F344 rats as an experimental model of photoperiodism is that they provide a good system for comparing the results with nonseasonal rodents, as seen in recent studies on the hypothalamic-pituitary-adrenal axis (24) and circadian rhythms (23), as well as on food selection and the mechanisms underlying energy metabolism (45).
